# Bovine Colostrum Supplementation Improves Bone Metabolism in an Osteoporosis-Induced Animal Model

**DOI:** 10.3390/nu13092981

**Published:** 2021-08-27

**Authors:** Eirini K. Kydonaki, Laura Freitas, Bruno M. Fonseca, Henrique Reguengo, Carlos Raposo Simón, Ana R. Bastos, Emanuel M. Fernandes, Raphaël F. Canadas, Joaquim Miguel Oliveira, Vitor M. Correlo, Rui L. Reis, Maria Vliora, Parakevi Gkiata, Yiannis Koutedakis, Georgia Ntina, Rui Pinto, Andres E. Carrillo, Franklim Marques, Tânia Amorim

**Affiliations:** 1 UCIBIO/REQUIMTE, Faculty of Pharmacy, University of Porto, 4050-313 Porto, Portugal; eir.kyd@gmail.com (E.K.K.); laura_c_freitas@hotmail.com (L.F.); brunofonseca@ff.up.pt (B.M.F.); hlreguengo@gmail.com (H.R.); franklim@ff.up.pt (F.M.); 2 SALURIS, 28108 Madrid, Spain; craposoc@gmail.com; 3 Department of Pharmacology, Pharmacognosy and Botany, Complutense University, 28040 Madrid, Spain; 4 3B’s Research Group, I3Bs—Research Institute on Biomaterials, Biodegradables and Biomimetics, University of Minho, Headquarters of the European Institute of Excellence on Tissue Engineering and Regenerative Medicine, AvePark, Parque de Ciência e Tecnologia, Zona Industrial da Gandra, 4805-017 Guimarães, Portugal; raquel.bastos@i3bs.uminho.pt (A.R.B.); efernandes@i3bs.uminho.pt (E.M.F.); raphael.canadas@i3bs.uminho.pt (R.F.C.); miguel.oliveira@i3bs.uminho.pt (J.M.O.); vitorcorrelo@i3bs.uminho.pt (V.M.C.); rgreis@i3bs.uminho.pt (R.L.R.); 5 ICVS/3B’s—PT Government Associate Laboratory, 4805-017 Guimarães, Portugal; 6 School of Sports and Exercise Sciences, University of Thessaly, 42100 Trikala, Greece; mvliora@gmail.com (M.V.); gkiata.vivi@gmail.com (P.G.); y.koutedakis@pe.uth.gr (Y.K.); 7 Faculty of Education, Health and Wellbeing, University of Wolverhampton, Walsall WS1 3BD, UK; 8 BME, Biomechanical Solutions, 43150 Karditsa, Greece; ntinageorgia10@gmail.com; 9 iMed.UL, Faculty of Pharmacy, University of Lisbon, 1649-003 Lisbon, Portugal; rapinto@ff.ulisboa.pt; 10 Joaquim Chaves Saúde, 1495-068 Miraflores Algés, Portugal; 11 Department of Exercise Science, Chatham University, Pittsburgh, PA 15232, USA; acarrillo@chatham.edu; 12 Move-Cor Inc., Pittsburgh, PA 15017, USA

**Keywords:** bovine colostrum, bone, osteoporosis, supplementation

## Abstract

Osteoporosis is characterized by bone loss. The present study aims to investigate the effects of bovine colostrum (BC) on bone metabolism using ovariectomized (OVX) and orchidectomized (ORX) rat models. Twenty-seven-week-old Wistar Han rats were randomly assigned as: (1) placebo control, (2) BC supplementation dose 1 (BC1: 0.5 g/day/OVX, 1 g/day/ORX), (3) BC supplementation dose 2 (BC2: 1 g/day/OVX, 1.5 g/day/ORX) and (4) BC supplementation dose 3 (BC3: 1.5 g/day/OVX, 2 g/day/ORX). Bone microarchitecture, strength, gene expression of VEGFA, FGF2, RANKL, RANK and OPG, and bone resorption/formation markers were assessed after four months of BC supplementation. Compared to the placebo, OVX rats in the BC1 group exhibited significantly higher cortical bone mineral content and trabecular bone mineral content (*p* < 0.01), while OVX rats in the BC3 group showed significantly higher trabecular bone mineral content (*p* < 0.05). ORX rats receiving BC dose 2 demonstrated significantly higher levels of trabecular bone mineral content (*p* < 0.05). Serum osteocalcin in the ORX was pointedly higher in all BC supplementation groups than the placebo (BC1: *p* < 0.05; BC2, BC3: *p* < 0.001). Higher doses of BC induced significantly higher relative mRNA expression of OPG, VEGFA, FGF2 and RANKL (*p* < 0.05). BC supplementation improves bone metabolism of OVX and ORX rats, which might be associated with the activation of the VEGFA, FGF2 and RANKL/RANK/OPG pathways.

## 1. Introduction

Osteoporosis is a skeletal disorder characterized by loss of bone tissue [1,2]. It has been associated with functional decline, decreased quality of life, and increased morbidity/mortality due to osteoporotic fractures [3,4,5] and it can affect both physically active and non-active individuals [6,7]. In 2010, the economic burden of osteoporotic fractures in the EU was approximately €37 billion, while, by 2050, the incidence of osteoporotic fractures is expected to rise by 240% in women and 310% in men worldwide compared to 1990 [8].

It has been reported that the use of existing pharmacological means for combating osteoporosis is decreasing [8,9], as patients fear the side effects of certain agents, such as bisphosphonates (BPs) [9,10]. Specifically, several adverse effects have been reported with the use of Denosumab [11] (a fully human monoclonal antibody that inhibits the receptor activator of the nuclear factor kappa-B ligand (RANKL)), including serious infections [12], osteonecrosis of the jaw [13], and atypical femur fracture [14]. Additionally, raloxifene administration has been associated with an increased risk of deep vein thrombosis and pulmonary embolism [15,16], whilst treatment with calcitonin, a synthetic polypeptide hormone, has been linked with cancer incidence [17,18]. Furthermore, the benefits of long-term treatment with teriparatide, a recombinant form of parathyroid hormone, have also been questioned, leading to its restricted use both in the US and the EU [19].

Non-pharmacological management of osteoporosis includes the maintenance of sufficient vitamin D and calcium concentrations [20,21]. When sunlight exposure and dietary intake of vitamin D and calcium are insufficient, supplementation is often recommended [22,23], even in athletic populations [24,25]. However, the non-pharmacological management of osteoporosis has also been associated with concerning side effects [26,27,28,29]. For instance, calcium supplementation has been associated with increased cardiovascular disease risk [30] and gastrointestinal side effects [31].

Bovine colostrum (BC) is a non-pharmacological option that may safely improve bone health. Studies specifically designed to determine BC constituents [32,33,34] have shown that BC contains several bioactive components, including various growth factors, immunoglobulins, leukocytes, antimicrobial elements and lactoferrin (LF) [32,35], which induce positive effects on bone metabolism [36,37,38,39,40], both in vivo and in vitro [41,42]. Furthermore, BC has certain bioactive components also involved in bone metabolism, such as colostrum basic protein [37], BC acid proteins [38,43], exosomes [39] and growth protein-colostrum fraction [40]. Yet the effects of BC supplementation on bone metabolism and the molecular pathways through which BC may interact with bones are fairly unclear. Therefore, the aims of this study were to (a) investigate the effects of BC supplementation on bone metabolism, and (b) identify the signaling pathways that may mediate bone metabolic processes induced by BC using an osteoporosis animal model. We report evidence on how BC supplementation affects the bone metabolism of ovariectomized (OVX) and orchidectomized (ORX) rats [44,45] and on which signaling pathways may be associated with these observed effects.

## 2. Materials and Methods

### 2.1. Bovine Colostrum Preparation

BC (collected during the first 24 h postpartum) was obtained from a local milk producer. Immediately after collection, BC was stored (−20 °C) for 72 h. Following lyophilization, BC was kept at room temperature in plastic zipper bags and in sealed polystyrene boxes embedded with Silica Gel Desiccant Beads to avoid humidity until it was used for oral supplementation.

### 2.2. Animal Care and Use

All animal procedures were carried out in accordance with the National and European guidelines for animal care and use; specifically, the EU directive 2010/63/EU. The study was approved by the National Ethics Committee for the Use of Animals in Research (ORBEA). Female and male Wistar Han rats were included in the present study. In order to induce osteoporosis, an ovariectomy (OVX, *n* = 32) was operated in female rats, and an orchidectomy in male rats (ORX, *n* = 32) as previously suggested [44,45,46]. Surgeries were performed at the age of 27 weeks under general anesthesia induced by sequential injections of buprenorphine (0.05 mg/kg body weight, i.p.), metoclopramide (1 mg/kg body weight, i.p.) and a solution of xylazine and ketamine (190 + 100 μL/200 g body weight, i.p.), maintained with a volatile anesthetic system of 3–4% isoflurane. After surgery, rats were placed in individual cages for 72 h (during the first 24 h, rats were kept in a recovery unit station with a temperature of 23 °C and a relative humidity of 45–55%). During recovery, all animals underwent an analgesic plan consisting of oral administration of paracetamol (25–400 mg/kg body weight), tramadol (5–20 mg/kg body weight) and metoclopramide (0.2–1 mg/kg) every 12 h. Following recovery, animals were allocated in pairs in conventional cages type III and IV with corncob bedding, under the vivarium conditions of a 12 h dark/light cycle, mean temperature of 22 ± 2 °C and a relative humidity of 55 ± 10%. All rats had ad libitum access to water and standard rodent feed.

### 2.3. Study Design

Thirty days following OVX and ORX surgeries, animals were randomly assigned to 1 of 4 groups: (1) placebo control (OVX, *n* = 8; ORX, *n* = 8), (2) BC supplementation dose 1 (BC1; OVX, *n* = 8; ORX, *n* = 8), (3) BC supplementation dose 2 (BC2; OVX, *n* = 8; ORX, *n* = 8) and (4) BC supplementation dose 3 (BC3; OVX, *n* = 8; ORX, *n* = 8) (Table 1). The following doses were used for four months: (1) the placebo group was given a cereal flour-based mash (0.5 g/day); (2) BC1 group (OVX: 0.5 g/day; ORX: 1 g/day), (3) BC2 group (OVX: 1 g/day; ORX:1.5 g/day) and (4) BC3 group (OVX: 1.5 g/day; ORX: 2 g/day). After the four-month supplementation period, all rats were euthanized; blood and bone samples were collected. The doses were determined based on a previous study [47]. The differences in the administrated BC dosage between OVX and ORX rats were due to variations in body weight.

### 2.4. Bone Biomarkers

Blood was collected post-supplementation—after euthanasia (total circulating blood volume; cardiac, cranial vena cava puncture). Samples were centrifuged, and the serum was separated and stored at −80 °C. Serum osteocalcin (OC), alkaline phosphatase (ALP), and deoxypyridinoline (D-Pyr) were assessed using ELISA kits (OC: Biorbyt; ALP: Mybiosource; D-Pyr: Mybiosource).

### 2.5. Bone Microarchitecture (MicroCT)

A high-resolution X-ray microtomography (Micro-CT) system (SkyScan 1272, Kontich, Belgium) was used to assess the morphometric parameters of the segmenting bones. Projections with 4 μm pixel size were acquired over a rotation range of 360° with a rotation step of 0.45° and an aluminum 0.25 mm filter. The 2D cross-sectional images were reconstructed using a standardized cone-beam reconstruction software (NRecon1.6.10.2, Bruker, Kontich, Belgium). A binary picture was created using at least 30 slides with a thresholding between 40 and 255 on a grey scale. A CT-analyzer program (CTAn, v1.17.0.0., SkyScan, Belgium) was utilized for 3D morphometric analysis. In order to calibrate bone mineral density (BMD) with Hounsfield units (HU), two hydroxyapatite [Ca_10_(PO_4_)_6_(OH)_2_] phantoms with BMD 0.250 and 0.750 g/cm^3^ were used. Cortical porosity (Ct.Pr), cortical object volume (Ct.OV), cortical BMD (Ct.BMD), cortical bone mineral content (Ct.BMC), trabecular porosity (Tb.Pr), trabecular separation (Tb.Sp), trabecular thickness (Tb.Th), trabecular object volume (Tb.OV), trabecular BMD (Tb.BMD), and trabecular BMC (Tb.BMC) were assessed.

### 2.6. Mechanical Properties

The biomechanical properties of the ORX and OVX rats’ tibias were examined using uniaxial tensile tests (adapted from [48,49,50]), using Instron 4505 Universal Mechanical Testing Equipment equipped with a BioPlus pneumatic tensile grips system (Instron, MA, USA). Prior to the assay the bones were stored in a room at 4 °C and in a formalin solution. The tibias were removed and washed with distilled water and placed in a phosphate buffered saline (PBS) solution for 2 h before the test. The mechanical tests were conducted using a 50 N load cell, a crosshead speed of 2 mm/min^−1^ and a distance between grips of 10 mm. Six specimens per condition were tested, including three tibia from rat females and three tibia from males. The elastic modulus (*Ε*) was determined from the initial slope in the stress–strain curve, and the stress and strain at yield (σy) as well as the maximum tensile strength (σ) were calculated using the Bluehill Universal software.

### 2.7. Gene Expression

Following euthanasia, left tibias were collected and stored in empty tubes at −80 °C for gene expression analysis. Gene expression of targeted genes (Table 2) was analyzed by quantitative real-time reverse transcription-PCR (qRT-PCR). RNA from each tibia was extracted by breaking the bone into small pieces using diagonal pliers. Bone pieces were further kept in prechilled potters containing 1 mL TripleXtractor reagent (grisp, Research Solutions, Porto, Portugal) followed by homogenization with a basic ULTRA-TURRAX for 1 min at full speed. RNA was further extracted according to the manufacturer’s protocol and quantified using a NanoDrop ND-1000 spectrophotometer (NanoDrop Technologies, Inc., Wilmington, DE, USA); quality was assessed by Experion (Bio-Rad Laboratories, USA). Two micrograms of RNA were reverse-transcribed using the Xpert cDNA synthesis kit (grisp, Research solutions, Portugal). This was performed by using the Xpert Fast SYBR Mastermix Kit (grisp, Research solutions, Portugal) in the Real-Time PCR Detection System (StepOnePlus, applied biosystems, USA), following the manufacturer’s protocol. PCR was initiated with a denaturation step at 95 °C for 3 min, followed by up to 40 cycles of denaturation, annealing and primer extension. Primer sequences and PCR conditions are listed in Table 2. The fold change in gene expression was calculated using the 2^−ΔΔCt^ method [51], with the housekeeping gene GAPDH as the internal gene, though the presented data were calculated by using the GAPDH gene normalized to the control group. RANKL:OPG ratios were further calculated from the relative mRNA levels of RANKL and OPG.

### 2.8. Statistical Analyses

The power analysis was based on a previous study with a similar design [52]. Assuming a detectable difference of a 0.4 standard deviation and 85% power, calculations indicated that a sample of seven rats per group was required.

Bone microarchitecture, mechanical testing, and blood biochemistry results are reported as mean ± standard deviation (SD), while gene expression results are reported as mean ± SEM. The Statistical Package for the Social Sciences (SPSS 26.0) software package was used. For the Micro-CT and blood biochemistry results, non-parametric tests were performed; the Mann–Whitney U test was used to compare outcome variables between groups (post-intervention). Statistical analysis for gene expression was performed using one-way ANOVA, followed by a Bonferroni ad hoc post-test to make pairwise comparisons of individual means using GraphPad Prism (version 8.1.2; GraphPad Software, Inc., San Diego, CA, USA). Differences were considered statistically significant when *p* < 0.05.

Effect size (d) values were calculated for bone microarchitecture, strength, and resorption/formation markers; effect size values were interpreted as none (0.0–0.19), small (0.2–0.49), medium (0.5–0.79), or large (≥0.8) [53].

## 3. Results

### 3.1. Bone Microarchitecture

Compared to the placebo, OVX rats receiving the lowest dose of BC (0.5 g/day) demonstrated higher cortical bone mineral content and trabecular bone mineral content (*p* < 0.01) (Table 3). Paradoxically, OVX rats in the BC1 group appeared to have significantly higher Ct.Pr (*p* < 0.01) and Tb.Pr (*p* < 0.05), but significantly lower Ct.OV (*p* < 0.01), Tb.OV (*p* < 0.05), cortical bone mineral content, trabecular bone mineral content (*p* < 0.01), and Tb.Th (*p* < 0.01) compared to placebo. There was a large effect of the second dose of BC supplementation (1 g/day) on cortical bone mineral content (*p* = 0.093, d = 1.02) and on trabecular bone mineral content (*p* = 0.189, d = 0.97) in OVX rats where along with the Ct.OV (*p* > 0.05) and Tb.OV (*p* > 0.05) they were found higher than the placebo. The highest dose of BC (1.5 g/day) revealed similar findings to those of the BC2 group in the OVX rats; i.e., trabecular bone mineral content was also significantly higher in the BC2 group compared to the placebo (*p* < 0.05). Regarding ORX rats receiving the lowest dose of BC (1 g/day), Ct.Pr was significantly higher, whereas Ct.OV was significantly lower following BC supplementation (compared to the placebo group) (*p* < 0.05). Even though it did not reach statistical significance, trabecular bone mineral content (*p* > 0.05) appeared to be higher in the BC1 group following supplementation compared to the placebo. ORX rats in the BC2 group presented significantly higher trabecular bone mineral content compared to the placebo following BC supplementation (*p* < 0.05). Moreover, in the same group of supplementation, Ct.Pr (*p* = 0.141, d = 0.37), Tb.Pr (*p* = 0.115, d = 0.66), and Tb.Sp (*p* = 0.753, d = 0.34) presented lower values following supplementation, whereas Ct.OV (*p* = 0.141, d = 0.38), Tb.OV (*p* = 0.115, d = 0.66), cortical bone mineral content (*p* = 0.115, d = 0.80), cortical bone mineral content (*p* = 0.248, d = 0.13) and Tb.Th (*p* = 0.529, d = 0.50) presented higher values compared to the placebo. There was no difference between rats of the BC3 group and rats of the placebo group in any parameter.

### 3.2. Mechanical Properties

In this work, the biomechanical properties of the ORX and OVX rats’ tibias were measured under tensile load (Table 4). The maximum tensile strength (σ), which corresponds to the maximum force of the stress–strain curve, for placebo ORX rats was 3.84 ± 0.63 MPa and for OVX rats it was 8.00 ± 0.75 MPa. With BC supplementation, the strength values ranged between 4.36 ± 0.90 and 6.22 ± 1.74 MPa. Comparing the placebo condition with the increase of BC supplementation dose, a small increase in the tensile strength properties was observed, which might correspond to a reinforcement on the biomechanical properties of the rat tibia. The stiffness of the material was also determined and is indicated by the elastic modulus (*E*), which corresponds for ORX rats to 151.77 ± 35.31 MPa and for OVX rats to 385.06 ± 54.14 MPa. In both properties, mechanical values were higher for OVX rats compared to the ORX rat tibias. Moreover, and comparing the placebo tibias with the remaining conditions of BC supplementation, significant statistical differences were not observed (*p* > 0.05). However, in the BC1, BC2, and BC3 groups, the difference between ORX and OVX rats are significantly reduced, with some increase in the mechanical performance for the ORX rat specimens, suggesting a positive effect after the BC supplementation. We also determined the point of transition between the elastic area and the plastic area of the tensile curve, which is called the yield point, which corresponds to the yield stress or maximum elastic resistance (σy) and to the yield strain (εy), which estimates the capacity of the bone to become strained without suffering micro-fractures. Once again, the yield stress for OVX rat tibias was higher compared to the ORX rat conditions, ranging in mean values from 2.17 up to 3.53 MPa for OVX rats and 1.88 up to 2.50 MPa for the ORX rats’ tibia conditions. As expected, regarding this property, the values were in the same range for all of the conditions and no significant differences between the groups was observed.

### 3.3. Bone Biomarkers

Table 5 shows the results obtained for bone biomarkers after BC supplementation. Serum D-Pyr was found to be lower in the OVX rats of the BC1 group (*p* = 0.385, d = 0.43) and BC3 group (*p* = 0.269, d = 0.64) compared to the placebo group. Furthermore, there was a trend indicating higher OC (*p* = 0.058, d = 0.90) in the OVX rats receiving the third dose of BC (1.5 g/day) compared to the placebo group. Serum levels of OC in the ORX rats were found to be significantly higher in all three groups of BC supplementation compared to the placebo group (*p* < 0.05; *p* < 0.001, respectively) following BC supplementation. Moreover, ORX rats supplemented with the third dose of BC (2 g/day) revealed higher serum ALP (*p* = 0.529, d = 0.29) levels and lower serum D-Pyr (*p* = 0.223, d = 0.80) levels compared to the placebo. Serum D-Pyr was found to be significantly higher in ORX rats of the BC1 group compared to the placebo (*p* < 0.05).

### 3.4. Gene Expression

**Figure 1:**
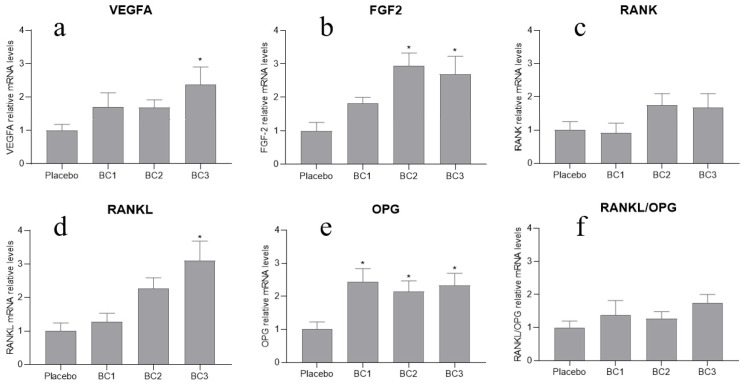
qPCR analysis for VEGFA, FGF2, RANK, RANKL and OPG. (**a**) qPCR for VEGFA mRNA; (**b**) qPCR for FGF2 mRNA; (**c**) qPCR for RANK mRNA; (**d**) qPCR for RANKL mRNA; (**e**) qPCR for OPG mRNA; (**f**) RANKL/OPG ratio calculated by the relative mRNA values of RANKL and OPG. Data are mean ± SD. Significant differences from the control (placebo supplementation group) are presented by * (*p* < 0.05).

As shown in Figure 1, the local expression of the FGF2 gene was higher in the BC2 and the BC3 supplementation groups compared to the placebo group (*p* < 0.05; Figure 1b). Regarding VEGFA gene, only BC dose 3 (1.5 g/day/OVX rats, 2 g/day/ORX rats) induced a significantly higher expression of VEGFA compared to the placebo (*p* < 0.05; Figure 1a). Similar findings were found in relation to RANKL gene expression; only the highest dose of BC (1.5 g/day/OVX rats, 2 g/day/ORX rats) induced higher local expression of RANKL compared to the placebo (*p* < 0.05; Figure 1d). Moreover, OPG mRNA expression was statistically higher in all BC supplementation groups compared to the placebo (*p* < 0.05; Figure 1e). We found no statistically significant changes in RANKL/OPG ratio in any of the groups supplemented with the three different doses of BC (*p* > 0.05; Figure 1f).

## 4. Discussion

We found that BC supplementation improved bone parameters (i.e., both cortical and trabecular bone) in an adult rat model of osteoporosis (OVX and ORX) in a dose-dependent manner. Specifically, at the highest doses of BC (1 g/day/OVX rats, 1.5 g/day/ORX rats, and/or 1.5 g/day/OVX rats, 2 g/day/ORX rats) we observed that both cortical and trabecular bones improved in both OVX and ORX rats (as well as the bone formation marker OC). Furthermore, we provide evidence regarding the signaling pathways stimulated in bones with BC supplementation, as our results indicate that VEGFA, FGF2, and RANKL/RANK/OPG pathways may be associated with the bone anabolic effects observed in this study, induced by BC supplementation. Our findings also demonstrate the potential of BC supplementation to enhance intrinsic bone material properties as suggested by the mechanical testing results. Regarding the later, BC appeared to have a dose-effect in some of the mechanical properties of the OVX rats as well, as it was shown to have higher σy and εy values in the higher supplemented doses of BC (1 g/day and 1.5 g/day/OVX rats). For the σ and Ε, however, both the lowest and highest administered doses of BC (0.5 and 1.5 g/day) were found to be the most favorable to induce bone strength and stiffness in OVX rats. These findings suggest that BC supplementation has the potential to improve bone mechanical properties by improving bone strength and stiffness, while reducing bone brittleness, resulting in bones that may be less susceptible to fractures.

To further understand how BC supplementation may affect bones’ remodeling cycle, serum ALP and OC (as indicative of bone formation), and serum D-Pyr (as indicative of bone resorption) were measured. Serum OC levels in our ORX rats receiving BC supplementation significantly increased in a dose-dependent manner; i.e., 2 g/day of BC induced the biggest increase in OC, which indicates that BC may be stimulating osteoblast activity [54]. Medium and large effects were found for the highest supplemented dose of BC on D-Pyr in OVX (1.5 g/day) and ORX (2 g/day) rats, respectively, which makes it reasonable to suggest that BC may inhibit bone resorption; however, further research needs to be carried out to confirm this claim.

BC contains several components that have been associated with bone metabolism, such as the lactoferrin (LF). In vitro studies have shown that LF stimulates osteoblast differentiation and proliferation [41,42,55,56] and decreases osteoblast apoptosis [57]. Moreover, it has been shown that LF also inhibits differentiation of osteoclasts [41,58,59] and reduces their resorbing activity [47,52,58,60,61,62,63]. In vivo, oral administration of LF has been shown to improve bone mass, microarchitecture, biomechanical, and strength parameters in OVX mice [58,62] and rats [47,52,61]. In a randomized controlled trial, postmenopausal women receiving an RNA-se enriched LF supplementation improved bone-specific formation markers (ALP increased by 45% and OC by 16%), while reducing bone-specific resorption markers (urine D-Pyr decreased by 14%) [63]. Therefore, it is reasonable to assume that the positive effects induced by BC in the present study are due to LF, which is in line with available data indicating that lactoferrin (one of the main BC component) supplementation increased levels of serum OC in OVX rats [62].

The mechanisms by which BC affects bone metabolism are currently unknown. The RANKL/RANK/OPG signaling pathway is a possible candidate due to its important role in the regulation of bone resorption [64]. RANKL is part of the tumor necrosis factor (TNF) family and is known to regulate the activation, development, differentiation, and maintenance of osteoclasts [65,66,67]. Furthermore, osteoclastogenesis and activation, differentiation and survival of the osteoclasts takes place when RANKL binds to its receptor RANK [65,68,69]. OPG is a member of the TNF receptor super-family (TNFRS) and also is a decoy receptor of RANKL, which results in blocking the binding between RANK and RANKL. Thus, OPG inhibits the effects that RANK and RANKL have on osteoclasts when binding together [70,71] (e.g., osteoclastogenesis) resulting in a protective role against bone loss and osteoporosis [72,73]. We found that all administered doses of BC induced a higher OPG gene expression in the tibia. Furthermore, the relative mRNA expression of RANKL significantly increased at the highest administered dose of BC (1.5 g/day/OVX; 2 g/day/ORX).

Due to the importance of the relationship between the RANKL and the OPG in osteo-clast biology, we further calculated the RANKL/OPG ratio; we found that our BC did not induce any statistically significant change in the RANKL/OPG ratio. These unexpected results may have been due to biphasic effects whereby BC treatment stimulates RANKL only at high doses but stimulates OPG at all assayed concentrations. Therefore, no significant changes were observed in the RANKL/OPG ratio. These contentions require further examination, yet the higher OPG results in combination with the statistically unaffected RANK relative mRNA levels suggest that there may be an inhibitory effect on osteoclastogenesis. Furthermore, our results suggest that the RANKL/RANK/OPG signaling pathway may be an important starting point for future research investigating the mechanisms by which BC affects bone metabolism.

Other possible signaling pathways by which BC may affect bone metabolism are through the angiogenic factors VEGFA and FGF2. VEGFA has been found to stimulate differentiation and migration of osteoblasts in vitro [74,75,76] and play an important role in bone development and regeneration in vivo [77,78,79]. FGF2 has also been found to stimulate (a) bone formation in vivo [80,81,82], and (b) osteoblast differentiation and proliferation in vitro [83,84]. Interestingly, a decrease in bone mass and bone formation has been observed in FGF2 knock-out mice [85]. In the present study, the highest administered doses of BC (1 g/day/OVX; 1.5 g/day/ORX and 1.5 g/day/OVX; 2 g/day/ORX) triggered a higher local expression of the FGF2 gene in the tibia. Furthermore, the highest dose of BC (1.5 g/day/OVX; 2 g/day/ORX) promoted the highest relative mRNA expression of VEGFA. These results indicate that BC may be stimulating osteoblast differentiation, increasing bone growth and regeneration through the VEGFA and the FGF2 signaling. Future studies should further explore BC effects using both in vitro and in vivo models.

A recently published systematic review investigated the health benefits of colostrum supplementation in humans [86], where only one study focused on the effects of BC supplementation on bone health [87]. In the latter study, however, the participants were also performing resistance exercise during the supplementation period, which reinforces the need for studies that focus specifically on the relationship between BC supplementation and bone health. Considering the dearth of published data, results from the present study may be used to (1) guide future studies investigating the mechanisms by which BC affects bone, and (2) help design human BC intervention studies. The rat models used in the current study have been previously utilized to investigate the effectiveness of osteoporosis drugs that have already been translated into clinical practice (e.g., bisphosphonates and estrogens) [44]. Future studies in humans should focus on the proof of concept (i.e., phase 1 clinical trials) in both healthy individuals and osteoporosis patients.

It is reasonable to assume that the present study might have been influenced by methodological limitations. For instance, the absence of mineral homeostasis assessments, such as calcium and phosphate, as well as measurements of BC constituents. Moreover, the duration of the BC supplementation that took place in the present study might have been insufficient to produce statistically significant results in all of the measured variables. Furthermore, although power calculations were completed and the sample size was considered sufficient, we acknowledge that the number of rats in each group was rather small. Future studies should consider increasing the sample size and include a healthy control group.

## 5. Conclusions

In conclusion, BC supplementation seemed to improve the bone mass and bone microarchitecture of OVX and ORX rats by stimulating bone formation in a dose-dependent manner. Some of the observed positive effects of BC on bone metabolism might be associated with the activation of the VEGFA, FGF2, and RANKL/RANK/OPG pathways.

## Figures and Tables

**Table 1:** Project timeline.

Pre-Intervention	4 Months BC Supplementation	Post-Intervention
Surgery: Ovariectomy and orchidectomy	**Placebo**: 0.5 g/day/OVX/ORX rats	**Euthanasia**: Blood collection: bone formation and resorption markers. Right tibia removal: micro-CT, mechanical testing. Left tibia removal: gene expression.
	**BC dose 1**: 0.5 g/day/OVX rats 1.0 g/day/ORX rats	
	**BC dose 2**: 1.0 g/day/OVX rats 1.5 g/day/ORX rats	
	**BC dose 3**: 1.5 g/day/OVX rats 2.0 g/day/ORX rats	

OVX = ovariectomized rats; ORX = orchidectomized rats; Micro-CT = micro computed tomography; BC = bovine colostrum.

**Table 2:** Primer sequences for control and target genes.

Gene	Primers (5′-3′)	Conditions
FGF2	CAAAACCTGACCCGATCCCT	95 °C, 3 s
	AGAATCTGTCCCGTTCGGC	62 °C, 20 s
		72 °C, 15 s
VEGF-A	GCAGCGACAAGGCAGACTA	95 °C, 3 s
	GAGTGAAGGAGCAACCTCTCC	64 °C, 20 s
		72 °C, 15 s
OPG	AGGGCATACTTCCTGTTGCC	95 °C, 3 s
	CACAGCACAGCCACTTGTTC	62 °C, 20 s
		72 °C, 15 s
RANKL	ATTGTCCAGTCGCACTTCGT	95 °C, 3 s
	AGTCGAGTCCTGCAAACCTG	62 °C, 20 s
		72 °C, 15 s
RANK	TGGCCCGGATGAATACTTGG	95 °C, 3 s
	GCACACTGTGTCCTTGTTGAG	63 °C, 20 s
		72 °C, 15 s
TATA	AAGGTTCCCTCCTCTGCACT	95 °C, 3 s
	TGTACAGGTGGCTTGAACACT	62 °C, 20 s
		72 °C, 15 s
GAPDH	CTATAAATTGAGCCCGCAGCC	95 °C, 3 s
	CCTTCCCCATGGTGTCTGAG	55 °C, 20 s
		72 °C, 15 s
B-actin	TTTCTGCGCAAGTTAGGTTTT	95 °C, 3 s
	TTTCTGCGCAAGTTAGGTTTT	60 °C, 20 s
		72 °C, 15 s

**Table 3:** Bone microarchitecture post BC supplementation.

	Post-Intervention			
Analyzed Parameter	Placebo	BC1	BC2	BC3
Cortical bone				
Porosity (%)				
ORX rats	29.48 ± 4.24	39.56 ± 15.47 *	25.51 ± 13.64	25.91 ± 7.39
OVX rats	26.56 ± 11.14	68.03 ± 14.32 **	25.16 ± 8.83	25.22 ± 8.54
Volume (% BV/TV)				
ORX rats	70.43 ± 4.13	60.44 ± 15.47 *	74.49 ± 13.64	74.09 ± 7.39
OVX rats	73.36 ± 11.15	31.97 ± 14.32 **	74.84 ± 8.83	74.78 ± 8.54
BMD (g/cm^3^)				
ORX rats	2.84 ± 0.33	2.56 ± 0.91	3.17 ± 0.44	2.93 ± 0.37
OVX rats	2.93 ± 0.29	1.29 ± 0.63 **	2.33 ± 0.73	2.83 ± 0.31
BMC (g)				
ORX rats	71.97 ± 12.94	71.43 ± 11.41	74.53 ± 23.13	78.93 ± 10.89
OVX rats	71.21 ± 9.65	88.01 ± 7.50 **	74.99 ± 17.55	71.78 ± 14.76
Trabecular bone				
Porosity (%)				
ORX rats	87.17 ± 4.14	89.56 ± 3.13	84.23 ± 4.26	84.92 ± 4.22
OVX rats	87.21 ± 1.97	92.47 ± 3.85 *	86.62 ± 4.81	85.26 ± 2.24
Separation (µm)				
ORX rats	113.02 ± 96.58	150.04 ± 51.30	86.44 ± 38.52	77.91 ± 22.43
OVX rats	163.12 ± 86.59	179.55 ± 50.98	145.79 ± 91.25	104.57 ± 47.40
Thickness (µm)				
ORX rats	16.44 ± 1.40	15.45 ± 2.42	17.91 ± 3.66	16.78 ± 1.55
OVX rats	25.73 ± 20.54	12.65 ± 3.64 **	21.11 ± 3.28	18.39 ± 2.45
Volume (% BV/TV)				
ORX rats	12.82 ± 4.14	10.44 ± 3.13	15.77 ± 4.26	15.08 ± 4.22
OVX rats	12.78 ± 1.97	7.53 ± 3.85 *	13.75 ± 4.93	14.74 ± 2.24
BMD (g/cm^3^)				
ORX rats	1.23 ± 0.20	1.15 ± 0.19	1.29 ± 0.22	1.27 ± 0.25
OVX rats	1.19 ± 0.12	1.07 ± 0.09 **	0.97 ± 0.28	1.22 ± 0.12
BMC (g)				
ORX rats	64.81 ± 12.86	66.25 ± 17.12	78.61 ± 10.60 *	74.19 ± 10.19
OVX rats	61.23 ± 10.18	94.98 ± 12.48 **	71.59 ± 19.31	72.71 ± 13.40 *

QCT analyses were made in the right posterior limb. Values are mean ± SD. Non-parametric tests were used to compare groups. Statistical significance was set at 0.05. * *p* < 0.05; ** *p* < 0.01, significant difference from the placebo. BC1 = 0.5 g/day/OVX rats, 1 g/day/ORX rats; BC2: 1 g/day/OVX rats, 1.5 g/day/ORX rats; BC3: 1.5 g/day/OVX rats, 2 g/day/ORX rats; BMD = bone mineral density; BMC = bone mineral content.

**Table 4:** Mechanical properties of the ORX and OVX rats’ tibias.

	Post-Intervention			
Analyzed Parameter	Placebo	BC1	BC2	BC3
Max. tensile strength (σ, MPa)				
ORX rats	3.84 ± 0.63	4.36 ± 0.90	5.00 ± 0.64	6.00 ± 0.45
OVX rats	8.00 ± 0.75	5.04 ± 0.76	4.86 ± 1.02	6.22 ± 1.74
Elastic modulus (E, MPa)				
ORX rats	151.77 ± 35.31	147.79 ± 9.30	192.33 ± 36.19	239.05 ± 21.42
OVX rats	385.06 ± 54.14	254.25 ± 53.54	202.02 ± 5.58	277.45 ± 74.13
Stress at yield (σy, MPa)				
ORX rats	1.88 ± 0.34	1.98 ± 0.78	2.39 ± 0.36	2.50 ± 1.03
OVX rats	3.53 ± 0.46	2.17 ± 0.44	2.83 ± 1.86	3.11 ± 0.46
Strain at yield (εy, %)				
ORX rats	1.37 ± 0.22	1.47 ± 0.45	1.39 ± 0.15	1.21 ± 0.43
OVX rats	1.10 ± 0.02	1.09 ± 0.31	1.45 ± 0.50	1.32 ± 0.26

Values are mean ± SD. Non-parametric tests were used to compare groups. Statistical significance was set at 0.05. BC1 = 0.5 g/day/OVX rats, 1 g/day/ORX rats; BC2: 1 g/day/OVX rats, 1.5 g/day/ORX rats; BC3: 1.5 g/day/OVX rats, 2 g/day/ORX rats; max. = maximum.

**Table 5:** Bone biomarkers post BC supplementation.

	Post-Intervention			
Analyzed Parameter	Placebo	BC1	BC2	BC3
Alkaline phosphatase (U/L)				
ORX rats	114.50 ± 10.74	103.14 ± 14.01	97.03 ± 17.19 *	119.73 ± 21.86
OVX rats	92.08 ± 26.29	72.83 ± 19.93	75.76 ± 26.08	70.8 ± 19.87
Osteocalcin (µg/L)				
ORX rats	10.71 ± 0.58	12.47 ± 1.44 *	13.74 ± 1.51 **	16.58 ± 1.54 **
OVX rats	13.35 ± 2.47	12.24 ± 1.14	11.09 ± 1.58	15.59 ± 2.24
Deoxypyridinoline (µg/L)				
ORX rats	0.44 ± 0.04	0.49 ± 0.05 *	0.45 ± 0.13	0.37 ± 0.11
OVX rats	0.43 ± 0.16	0.37 ± 0.09	0.44 ± 0.10	0.34 ± 0.10

Values are mean ± SD. Non-parametric tests were used to compare groups. Statistical significance was set at 0.05. * *p* < 0.05; ** *p* < 0.001, significant difference from the placebo. BC1 = 0.5 g/day/OVX rats, 1 g/day/ORX rats; BC2: 1 g/day/OVX rats, 1.5 g/day/ORX rats; BC3: 1.5 g/day/OVX rats, 2 g/day/ORX rats.

## Data Availability

The datasets generated during and/or analyzed during the current study are available from the corresponding author upon a reasonable request.
